# Expression of the luteinizing hormone receptor (LHR) in ovarian cancer

**DOI:** 10.1186/s12885-019-6153-8

**Published:** 2019-11-15

**Authors:** Shigang Xiong, Paulette Mhawech-Fauceglia, Denice Tsao-Wei, Lynda Roman, Rajesh K. Gaur, Alan L. Epstein, Jacek Pinski

**Affiliations:** 1 Department of Medicine/Medical Oncology Division, University of Southern California, 1441 Eastlake Ave, Los Angeles, CA 90033 USA; 2 Aurora Diagnostics, Department of Pathology, Gynecologic Pathology Consultant, San Antonio, TX 78209 USA; 3 University of Southern California, Norris Comprehensive Cancer Center, 1441 Eastlake Avenue, Los Angeles, CA 90033 USA; 4 Department of Obstetrics & Gynecology, University of Southern California Keck School of Medicine, Los Angeles, CA 90033 USA; 5 Department of Pathology, University of Southern California, HMR 2011 Zonal Ave, Los Angeles, CA 90033 USA

## Abstract

We investigated the association of LHR expression in epithelial ovarian cancer (OC) with clinical and pathologic characteristics of patients. LHR expression was examined immunohistochemically using tissue microarrays (TMAs) of specimens from 232 OC patients. Each sample was scored quantitatively evaluating LHR staining intensity (LHR-I) and percentage of LHR (LHR-P) staining cells in tumor cells examined. LHR-I was assessed as no staining (negative), weak (+ 1), moderate (+ 2), and strong positive (+ 3). LHR-P was measured as 1 to 5, 6 to 50% and >  50% of the tumor cells examined. Positive LHR staining was found in 202 (87%) patients’ tumor specimens and 66% patients had strong intensity LHR expression. In 197 (85%) of patients, LHR-P was measured in > 50% of tumor cells. LHR-I was significantly associated with pathologic stage (*p* = 0.007). We found that 72% of stage III or IV patients expressed strong LHR-I in tumor cells. There were 87% of Silberberg’s grade 2 or 3 patients compared to 70% of grade 1 patients with LHR expression observed in > 50% of tumor cells, *p* = 0.037. Tumor stage was significantly associated with overall survival and recurrence free survival, *p* < 0.001 for both analyses, even after adjustment for age, tumor grade and whether patient had persistent disease after therapy or not. Our study demonstrates that LHR is highly expressed in the majority of OC patients. Both LHR-I and LHR-P are significantly associated with either the pathologic stage or tumor grade.

## Background

Ovarian cancer (OC) remains the leading cause of death among gynecological malignancies, representing 239,000 patients and resulting in 152,000 deaths every year globally [1]. There is an urgent need to identify prognostic factors in order to better understand the pathogenesis of this deadly disease. The ovaries represent a chief part of the female reproductive system and target for the pituitary hormone, luteinizing hormone (LH). Prior to ovulation, LH triggers a cascade of fundamental events in cell meiosis, mitosis, differentiation, proliferation in ovarian tissue, such as resumption of meiosis of the oocyte, cumulus expansion, rupture of the follicular wall, and extrusion of the cumulus–oocyte mass [2]. Several clinical and epidemiologic studies have implicated reproductive changes with increased risk of OC which has been associated with menopause [3], the use of fertility drugs [4], and infertility and nulliparity [5]. Moreover, high levels of LH were consistently found in malignant effusions, such as ascites or cystic fluids of OC, as compared to those of non-malignant ovarian tumor origins [6, 7]. These observations have led to the hypothesis that pituitary-gonadal signaling may be involved in the carcinogenesis or progression of OC [8].

LH and human chorionic gonadotropin (hCG) bind to a common transmembrane glycoprotein receptor LHR (or LHCGR), a member of the G protein-coupled receptor family [9], resulting in activation of adenyl cyclase and cAMP production [10]. The expression of LHR mRNA [11], protein, and LHR binding activity [12] have been characterized in OC and ovarian surface epithelium, the putatively histogenetic origin of the most OCs. Mandai et al. [13] documented expression of LHR mRNA in 55.3% (26 of 47) of OC patient tissue samples while Lenhard et al. showed LHR protein expression by immunohistochemistry in 64.3% of OC cases [14]. Employing in situ hybridization and RT-PCR methods, Lu et al. [15] detected LHR expression in 42% of benign, 24% of borderline, and 17% of malignant ovarian tumors.

Although most studies show positive LHR expression in OC, data on the levels of expression and the role of this receptor in cancer progression are conflicting, limited, and, therefore, require further investigation. In this study, we assessed and quantified the concentration of LHR in a tissue microarray obtained from a large series of patients with OC who received treatment at our institution between 1991 and 2012 and evaluated the association of the LHR expression with clinical and pathologic characteristics of these patients.

## Methods

### Patients and specimens

Following approval by the Institutional Review Board (IRB), OC patients treated from 1991 to 2012 at the University of Southern California were found in our institutional archives and databases. Patient tissue specimens collected and medical records were collected and retrospectively reviewed under the approved IRB protocol. Patients’ age at diagnosis, pathologic stage and grade, outpatient and inpatient treatments, as well as patients’ survival and recurrence status, and follow-up information were documented for this study. The tumor histologic subtypes and grade were re-assessed on the hematoxylin-eosin (H&E) slides for confirmation by a single experienced pathologist (PMF). The Silverberg grading system was used as the tumor grading system [16].

### Tissue microarray construction

OC tissue microarrays (TMAs) were constructed utilizing archival tissue from eligible patients as described previously [17]. Briefly, A morphologically representative region was carefully selected from the chosen individual paraffin-embedded blocks of OC (donor blocks), followed by a 0.6 mm core tissue punch biopsy and subsequent transfer to the donor paraffin-embedded block (receiver block). To overcome tumor heterogeneity and tissue loss, 3 core biopsies were performed and extracted from different areas of each tumor. One section was stained with H&E to evaluate the presence of the tumor by light microscopy.

### Immunohistochemistry (IHC) for LHR expression

The monoclonal anti-human LHR antibody was prepared as described previously [18, 19] by Dr. Epstein’s laboratory at the University of Southern California. Briefly, the cDNA encoding the human LHR signal and extracellular domains was amplified and fused to the Fc region of human IgG1 by PCR assembling method. The fusion gene then was inserted into the *Hind 3* and *EcoR1* sites of expression vector pEE12, resulting in expression vector pEE12/LHR-Fc. The LHR-Fc fusion protein was expressed in NS0 murine myeloma cells for long-term stable expression in accordance with the manufacturer’s protocol (Lonza Biologics, Portsmouth, NH). The highest producing clone was scaled up for incubation in an aerated 3-L stir flask bioreactor using 5% dialyzed fetal calf serum (Lonza Biologics, Inc). The fusion protein was then purified from the filtered spent culture medium via tandom Protein-A affinity and ion exchange chromatography. The fusion protein was analyzed by SDS-PAGE to demonstrate proper assembly and purity. Four-week-old BALB/c female mice were injected subcutaneously with recombinant LHR-Fc in complete Freund’s adjuvant. Two weeks later, the mice were re-inoculated as above except in incomplete adjuvant. Ten days later, the mice received a third intravenous inoculation of antigen, this time without adjuvant. Four days later, the mice were sacrificed and the splenocytes fused with 8-azaguanine-resistant mouse myeloma NS0 cells. Culture supernatants from wells displaying active cell growth were tested via ELISA. Positive cultures were subcloned twice using limiting dilution methods and further characterized by flow cytometry and IHC.

For immunohistochemical studies, 4 μm thick sections were deparaffinized with xylene and re-hydrated in graded ethanol solutions. Antibody staining was performed using an ImmPress™ Excel staining kit according to the manufacturer’s instructions (Vector Laboratories, Burlingame, CA). Briefly, antigen retrieval was carried out by treating the deparaffinized sections in citrate buffer (pH 6.0) in a steam-cooker for 20 min. The sections were then incubated 10 min with 3% H_2_O_2_ to quench endogenous peroxidase activity followed by blocking with a 2.5% normal horse serum for 30 min. The slides were then incubated overnight with the above described antibody against LHR (clone 5F4; 1 μg/ml) along with the horse anti-mouse secondary, then incubated for 45 min at room temperature. The 3,3′-diaminobenzidine (DAB) was used as a chromogen. Sections were counterstained with hematoxylin and cover slipped. Sections of normal human ovarian tissue was used as positive controls. Negative control slides were included in all assays prepared by staining with secondary antibody only (Additional file 1 and Additional file 2).

### LHR expression scoring

For assessment of LHR expression, the immunostained TMA slides were reviewed and scored by an expert gynecologic pathologist (PMF). A scale of 0–3 was used to express the extent of IHC reactivity based on the LHR staining intensity (LHR-I) (complete absence of staining, 0; weak staining, + 1; moderate, + 2; strong, + 3) and the percentage of LHR stained cells (LHR-P) detected in tumor cells examined (0, < 5%, 6–50% and 51–100%). All other staining patterns were considered negative. Cores were not evaluated if the core was lost, severely damaged, and/or did not have sufficient tumor cellularity. The reviewer was blinded to original histological diagnosis and other clinical data. LHR expression scoring was performed, twice per month, by the same pathologist (PMF).

### Statistical analysis

Standard descriptive statistics were used to summarize baseline and study results. Fisher’s exact test was used to test the association of demographics and baseline clinical characteristics with LHR-I and LHR-P detected in tumor cells. Overall survival (OS) was calculated from date of definitive surgery to date of death or latest follow-up. Recurrence free survival (RFS) was calculated from date of definitive surgery to date of recurrence or death from any causes whichever observed first. Kaplan-Meier plots were used to estimate the probabilities of OS and RFS. The associated 95% confidence intervals were calculated using Greenwood’s standard errors formula. Log-rank test was used for testing the association of LHR expression intensity and percent observed in tumor cells, as well as the baseline clinical characteristics with OS and RFS. Cox proportional-hazards model was applied for multivariable analysis. All reported *p* values were two-sided and *p* values < 0.05 were considered statistically significant.

## Results

### Clinical and pathologic characteristics of patients

A total of 232 patients diagnosed with primary OC were included in this study. Among these patients, the median age at diagnosis was 58 years (range, 26–89 years). The histologic subtypes were 69% serous carcinoma, 9% endometrioid adenocarcinoma, 7% clear cell carcinoma, 6% mucinous carcinoma, 6% mixed, and 3% others. The vast majority of these patients (*n* = 140, 60%) were pathologic stage III and most of them were Silberberg grade 3 (76%), (Table 1). The median duration of follow-up was 68.6 months (range, 0.6–173.3) with median overall survival for all patients of 44.0 months (95% CI, 39.7, 49.9). The median recurrence free survival was 26.3 months (95% CI: 20.9, 38.0).

**Table 1:** Demographics and baseline disease characteristics

Total Patients		232		100%	
Surgery done		08/02/91–12/13/12			
Age at Diagnosis					
< 60		130		56%	
≥ 60		102		44%	
Median (Range)		58 (26–89)			
Tumor Histology					
Serous Carcinoma		160		69%	
Endometrioid Adenocarcinoma		21		9%	
Clear Cell Carcinoma		17		7%	
Mixed		15		6%	
Mucinous Carcinoma		13		6%	
MMMT		4		2%	
Undifferentiated		2		1%	
Pathologic Stage					
I		50		22%	
II		18		8%	
III		140		60%	
IV		24		10%	
Silberberg’s Grade					
1		27		12%	
2		28		12%	
3		177		76%	
Received Chemotherapy					
No		16		7%	
Yes		216		93%	
Residual Disease					
No		113		49%	
Yes		119		51%	
Persistent Disease					
No		144		62%	
Yes		88		38%	
LHR-I					
Negative		30		13%	
Weak		24		10%	
Moderate		26		11%	
Strong		152		66%	
LHR-P					
0%		30		13%	
1–50%		4		2%	
> 50%		197		85%	
Missing/LHR Intensity Negative		1			
Tumor Histology	N	LHR Expression Intensity			
		Negative	Weak	Moderate	Strong
Serous Carcinoma	160	16 (10%)	19 (12%)	16 (10%)	109 (68%)
Endometrioid Adenocarcinoma	21	3 (14%)	2 (10%)	3 (14%)	13 (62%)
Clear Cell Carcinoma	17	0 (0%)	0 (0%)	4 (24%)	13 (76%)
Mixed	15	4 (27%)	1 (7%)	3 (20%)	7 (47%)
Mucinous Carcinoma	13	6 (46%)	2 (15%)	0 (0%)	5 (38%)
MMMT	4	1 (25%)	0 (0%)	0 (0%)	3 (75%)
Undifferentiated	2	0 (0%)	0 (0%)	0 (0%)	2 (100%)

### Association of LHR intensity (LHR-I) and percentage of LHR expression (LHR-P) with demographic and disease characteristics

**Fig. 1:**
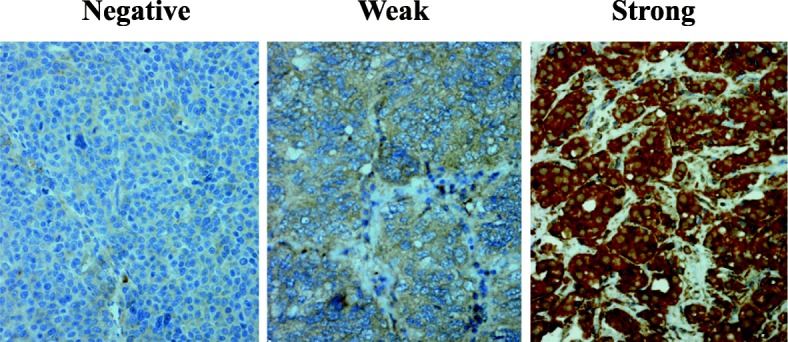
Expression of LHR protein in the specimens of primary epithelial OC on TMAs. Representative staining patterns of LHR immunohistochemical reactivity (negative, weak and strong) are presented (400×)

**Fig. 2:**
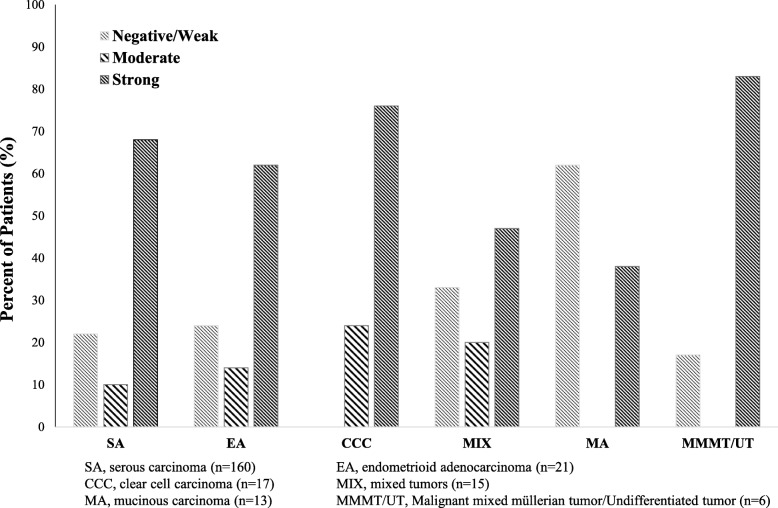
Distribution of LHR-I within Each Histology Groups. LHR is found to be strongly positive in 109/160 (68%) cases of serous carcinomas, 13/21 (62%) cases of endometrioid carcinomas, 13/17 (76%) cases of clear cell carcinoma, 5/13 (38%) cases of mucinous carcinoma, 7/15 (47%) cases of mixed tumors, and 5/6 (83%) cases of other types of carcinomas (inducing MMMT and undifferentiated tumors)

A total of 232 specimens of primary OCs on tissue microarrays (TMAs) were included in the IHC studies. Representative staining patterns (negative, weak, and strong staining) of LHR are illustrated in Fig. 1. The distribution of LHR-I within each histology group is shown in Fig. 2. As shown in Table 1, LHR was found to be strongly positive in 109/160 (68%) cases of serous carcinomas; 13/17 (76%) cases of clear cell carcinoma, 13/21 (62%) cases of endometrioid carcinomas, 5/13 (38%) cases of mucinous carcinoma, and 12/21 (57%) cases of other types of carcinomas. Among the 232 OC patients, 152 (66%) showed strong, 26 (11%) moderate, 24 (10%) weak staining, and 30 (13%) complete absence of staining (Table 1). LHR-I was significantly associated with pathologic tumor stage (*p* = 0.007). We found that 72% of stage III or IV patients expressed strong LHR-I in tumor cells (Table 2). From these data, 197 (85%) patients had more than 50% of the cancer cells stained positively for LHR (LHR-P) (Table 1). There were 87% of Silberberg’s grade 2 or 3 patients compared to 70% of grade 1 patients with LHR expression observed in cases positive with > 50% of tumor cells, *p* = 0.037 (Table 3).

**Table 2:** Association of LHR-I with demographics and disease characteristics

Factors	N	LHR Intensity				*p*-value*
		Negative	Weak	Moderate	Strong	
Age at Diagnosis						
< 60	130	21 (16%)	15 (12%)	16 (12%)	78 (60%)	0.22
≥ 60	102	9 (9%)	9 (9%)	10 (10%)	74 (73%)	
Serous Carcinoma						
No	72	14 (19%)	5 (7%)	10 (14%)	43 (60%)	0.13
Yes	160	16 (10%)	19 (12%)	16 (10%)	109 (68%)	
Pathologic Stage						
I/II	68	13 (19%)	8 (12%)	13 (19%)	34 (50%)	
III/IV	164	17 (10%)	16 (10%)	13 (8%)	118 (72%)	
Silberberg’s Grade						
1	27	7 (26%)	3 (11%)	2 (7%)	15 (56%)	0.21
2 or 3	205	23 (11%)	21 (10%)	24 (12%)	137 (67%)	
Persistent Disease						
No	144	22 (15%)	15 (10%)	17 (12%)	90 (63%)	0.53
Yes	88	8 (9%)	9 (10%)	9 (10%)	62 (70%)	

*p-value based on Fisher’s exact test

**Table 3:** Association of LHR-P with demographics and disease characteristics

Factors	N^a^	LHR Expression Observed in Tumor Cells		*p*-value*
		≤ 50%	> 50%	
Age at Diagnosis				
< 60	130	24 (18%)	106 (82%)	0.09
≥ 60	101	10 (10%)	91 (90%)	
Histology				
Serous	159	19 (12%)	140 (68%)	0.11
Other	72	15 (21%)	57 (79%)	
Pathologic Stage				
I/II	68	15 (22%)	53 (78%)	0.065
III/IV	163	19 (12%)	144 (88%)	
Silberberg’s Grade				
1	27	8 (30%)	19 (70%)	0.037
2 or 3	204	26 (13%)	178 (87%)	
Persistent Disease				
No	143	25 (17%)	118 (83%)	0.18
Yes	88	9 (10%)	79 (90%)	

^a^one patient doesn’t have LHR expression observed in tumor cell data available

*p-value based on Fisher’s exact test

### Association of overall survival and recurrence free survival with demographic and disease characteristics

Neither LHR intensity (LHR-I) nor the percent of LHR expressing tumor cells (LHR-P) were significantly associated with patient’s age at diagnosis, histologic subtypes (serous vs. others), or persistence of disease (Tables 2 and 3). OS and RFS were highly associated with tumor stage, even after adjustment for age at diagnosis, Silberberg’s grade, and whether the patient had persistent disease after therapy or not. No significant association was found between OS or RFS with LHR expression intensity (LHR-I) nor the percent of LHR positive tumor cells (LHR-P) (Table 4).

**Table 4:** Association of Overall Survival and Recurrence Free Survival with Demographics and Disease Characteristics

Factors	N	Overall Survival (Months)		Recurrence Free Survival (Months)	
		Median (95% CI)	*p*-value*	Median (95% CI)	*p*-value*
Overall	232	44.0 (39.7, 49.9)		26.3 (20.9, 38.0)	
Age at Diagnosis			< 0.001		< 0.001
< 60	130	52.2 (45.0, 84.9)	0.18^	38.0 (22.3, 49.9)	0.26^
≥60	102	36.8 (25.1, 41.8)		22.0 (15.3, 26.6)	
Pathologic Stage			< 0.001		< 0.001
I/II	68	Not reached	< 0.001^	Not reached	< 0.001^
III/IV	140	38.0 (30.1, 41.8)		20.0 (16.6, 24.2)	
Silberberg’s Grade			< 0.001		< 0.001
1	27	Not reached	0.23^	Not reached	0.060^
2 or 3	205	41.8 (37.9, 45.9)		24.2 (19.1, 29.9)	
Persistent Disease			< 0.001		< 0.001
No	144	69.8 (52.2, 85.7)	< 0.001^	35.8 (20.9, 51.4)	0.69^
Yes	88	24.2 (15.3, 30.5)		24.2 (15.3, 30.5)	
LHR-I			0.28		0.27
Negative/Weak/Moderate	80	51.2 (40.7, 65.9)		29.9 (19.0, 46.0)	
Strong	152	41.9 (38.0, 45.3)		24.5 (19.6, 38.0)	
LHR-P			0.36		0.53
≤ 50%	34	50.3 (35.4, 74.3)		36.0 (19.0, 50.3)	
> 50%	197	43.8 (39.2, 49.5)		24.6 (20.0, 38.0)	

*p-value based on logrank test

^ *p*-value based on Wald test from Cox proportional model, adjusted by all other variables with *p* < 0.05 in univariate analysis

## Discussion

Our results indicate that LHR is not only highly expressed, but also associated with advanced stages and tumor grade of OC. Previously, other groups have documented LHR expression in OC using different methods of measurement [12–14]. However, most of the aforementioned studies detected LHR in OC at lower concentrations by comparison to this study. This discrepancy could be owing to differences in the sensitivity and specificity of the LHR antibodies and detection kits used, and the associated sample sizes in those studies. Our results are based on a very large number of OC patients (232), allowing for a more representative distribution of histologic subtypes typically seen in the OC populations.

Gonadotropins and their receptor LHR have long been suggested to be involved in the progression of OC. Rapid growth of OC has been observed during early pregnancy when LH levels are high [20]. It has also been reported [6, 7] that significant concentrations of LH were measured in peritoneal and cystic fluids of women with OC. Moreover, a significant association was observed between high levels of LH and the degree of malignancy, indicating that gonadotropins may promote progression of LHR-positive OC. The incidence of OC has been shown to be increased under clinical conditions with elevated gonadotropins such as during menopause [3], infertility and nulliparity [5], or in women who receive induction treatment for ovulation [4, 21]. In contrast, reduced risk of OC was paired with clinical conditions associated with lower levels and reduced exposure to gonadotropins, such as multiple pregnancies, breast feeding, oral contraceptives, and estrogen replacement therapy [4, 5].

Several in vitro studies also support the stimulatory role of gonadotropins in the carcinogenesis and progression of OC. In studies with ovarian surface epithelium, a possible histogenetic origin of OC, treatment with hCG stimulated the proliferation of cells in a dose-dependent manner [12, 22]. Many in vitro studies on OC cell lines reported a stimulatory effect of LH/hCG on cell growth [23–25]. hCG stimulated (3H)-thymidine incorporation into DNA in LHR-expressing cells of normal ovarian surface epithelium (OSE) and the OC cell line OCC1, but not in LHR negative SKOV3 cells [24], suggesting that the stimulating effect of LH on OC is LHR-dependent. On the other hand, other groups of investigators demonstrated an inhibitory effect of LH on OC cell proliferation and release of CA-125 [26]. These conflicting findings could be explained by the different cell lines, in vitro conditions and concentrations of LH used in those studies. In addition to affecting OC cell proliferation, LH has also been shown to influence cellular processes, including adhesion [27], anchorage-independent growth [25], angiogenesis [28] and apoptosis [12, 23]. In animal models, OC could be induced after prolonged treatment with exogenous gonadotropins or elevated levels of endogenous gonadotropins [29]. In inhibin-alpha-deficient mice, gonadotropins were essential for gonadal and adrenal tumorigenesis [30], and chronically elevated circulating levels of LH or hCG caused ovarian and extragonadal tumors in certain strains of mice [31], strongly supporting the carcinogenic effect of gonadotropins on their target organs. LH is responsible for inducing ovulation in premenopausal women. The ovulatory process involves extensive proteolytic activity, cell proliferation, and tissue healing and remodeling, which parallels many cancer-associated processes [32].

Apoptosis is an important brake mechanism for carcinogenesis and cancer progression. It has been shown that hCG not only stimulates cell proliferation but also suppresses apoptosis in LHR-expressing cells of the OSE. This anti-apoptotic signaling of hCG was mediated by the insulin-like growth factor-1(IGF-1)/IGF-1 receptor pathway [12]. hCG treatment also demonstrated a LHR-dependent inhibition of cisplatin-induced apoptosis in LHR-positive OVCAR-3, but not in LHR-negative SK-OV-3 cells, suggesting a LHR-dependent inhibition via up-regulation of IGF-1. In addition, LH prevented cisplatin-induced apoptosis in oocytes [33]. During cyclic ovulation when the OSE is exposed to repeated injury and healing processes, apoptosis is likely to represent a protective mechanism by which injured cells are being eliminated. It is therefore possible that excessive stimulation of LH/hCG may enhance the susceptibility of OSE to carcinogenesis.

Despite the progress made with regard to diagnosis and treatment over the last years, OC remains a major cause of mortality [1]. Since expression of LHR can be found in most specimens, LH receptors might represent targets for immunotherapy or cytotoxic conjugated agents that can exploit these receptors to deliver hybridized cytotoxic moieties. Successful attempts have been made in animal experiments with hCG-hecate conjugates [34].

## Conclusions

Our study demonstrates that LHR is not only strongly expressed in the vast majority of OC specimens of different histology subtypes but it is also significantly associated with advanced tumor grades and pathologic stages of this disease. Further studies are needed to explore the role LHR in the carcinogenesis and progression of OC and to exploit the presence of this receptor as a target for novel therapies against OC.

## Supplementary information

**Additional file 1: Figure S1.** Western blot was performed with anti-LHR antibody (5F4) in HepG2 (positive) and LNCaP (positive) cell lines and were able to clearly show the about 85 kDa band for LHR expression. For the negative controls, CHO-K1 and DU145 cell lines were used, showing no LHR expression in both cell lines.

**Additional file 2: Figure S2.** Immunohistochemistry was performed with anti-LHR antibody (5F4) on the slides of normal human tissues (colon, liver and lung) as negative controls, showing no LHR immunoactivity in these tissues.

## Data Availability

All data and materials generated or analyzed during this study are included in this published article.

## Abbreviations

hCG: Human chorionic gonadotropin
IHC: Immunohistochemistry
LH: Luteinizing hormone
LHR: Luteinizing hormone receptor
LHR-I: LHR staining intensity
LHR-P: Percentage of LHR stained cells in tumor cells examined
OC: Ovarian cancer
OS: Overall survival
OSE: Normal ovarian surface epithelium
RFS: Recurrence free survival
TMAs: Tissue microarrays
