# Supplementary material for: Expression of the luteinizing hormone receptor (LHR) in ovarian cancer
Source: BMC Cancer. 2019 Nov 15;19:1114. doi: 10.1186/s12885-019-6153-8 (PMC6857310; doi:10.1186/s12885-019-6153-8)

**Supplemental Figure 1**. Western blot was performed with anti-LHR antibody (5F4) in HepG2 (positive) and LNCaP (positive) cell lines and were able to clearly show the about 85 kDa band for LHR expression. For the negative controls, CHO-K1 and DU145 cell lines were used, showing no LHR expression in both cell lines.


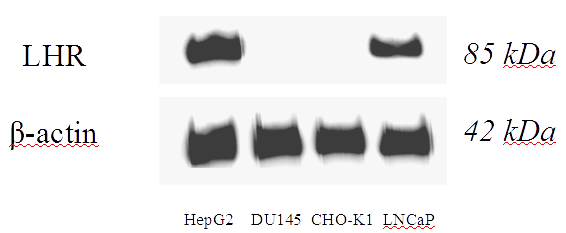

Supplement: Supplementary file 1 — Additional file 1: Figure S1. Western blot was performed with anti-LHR antibody (5F4) in HepG2 (positive) and LNCaP (positive) cell lines and were able to clearly show the about 85 kDa band for LHR expression. For the negative controls, CHO-K1 and DU145 cell lines were used, showing no LHR expression in both cell lines. [file 12885_2019_6153_MOESM1_ESM.docx]
