# Supplementary material for: Expression of the luteinizing hormone receptor (LHR) in ovarian cancer
Source: BMC Cancer. 2019 Nov 15;19:1114. doi: 10.1186/s12885-019-6153-8 (PMC6857310; doi:10.1186/s12885-019-6153-8)

**Supplemental Figure 2**. Immunohistochemistry was performed with anti-LHR antibody (5F4) on the slides of normal human tissues (colon, liver and lung) as negative controls, showing no LHR immunoactivity in these tissues.


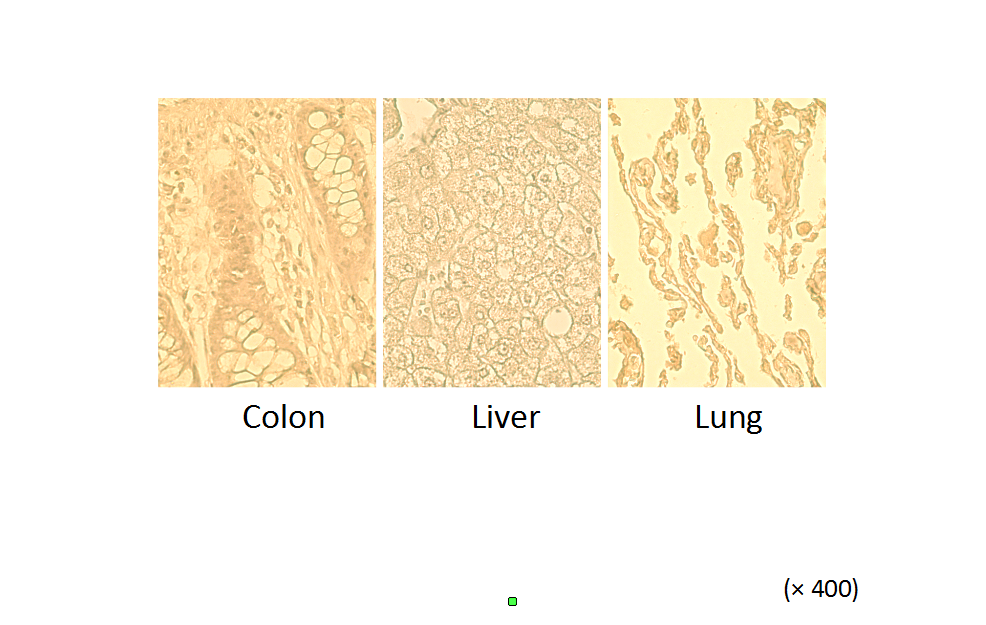

Supplement: Supplementary file 2 — Additional file 2: Figure S2. Immunohistochemistry was performed with anti-LHR antibody (5F4) on the slides of normal human tissues (colon, liver and lung) as negative controls, showing no LHR immunoactivity in these tissues. [file 12885_2019_6153_MOESM2_ESM.docx]
